# Phytochemical profile of Brazilian grapes (*Vitis labrusca* and hybrids) grown on different rootstocks

**DOI:** 10.1371/journal.pone.0275489

**Published:** 2022-10-20

**Authors:** Marlon Jocimar Rodrigues da Silva, Ana Paula Maia Paiva, Joyce Fagundes de Souza, Carla Valéria da Silva Padilha, Letícia Silva Pereira Basílio, Marcos dos Santos Lima, Giuliano Elias Pereira, Luiz Claudio Corrêa, Fabio Vianello, Giuseppina Pace Pereira Lima, Mara Fernandes Moura, Marco Antonio Tecchio

**Affiliations:** 1 Pernambuco State Agricultural Defense and Inspection Agency (ADAGRO), Petrolina, Pernambuco, Brazil; 2 Department of Horticulture, School of Agriculture, São Paulo State University (UNESP), Botucatu, São Paulo, Brazil; 3 Department of Technology and Social Sciences, Bahia State University (UNEB), Juazeiro, Bahia, Brazil; 4 Department of Food Technology, Federal Institute of Sertão Pernambucano, Petrolina, Pernambuco, Brazil; 5 Brazilian Agricultural Research Corporation (Embrapa Semiárido), Petrolina, Pernambuco, Brazil; 6 Department of Comparative Biomedicine and Food Science, University of Padova (UNIPD), Padova, Italy; 7 Department of Chemical and Biological Sciences, Institute of Biosciences, São Paulo State University (UNESP), Botucatu, SP, Brazil; 8 APTA Fruit Center, Agronomic Institute (IAC), Jundiaí, São Paulo, Brazil; Lincoln University, NEW ZEALAND

## Abstract

Important factors may influence the bioactive compounds in grapes, including scion–rootstock interaction. Therefore, the bioactive compounds and antioxidant activity in grape skin and pulp fractions of ‘Isabel Precoce’, ‘BRS Carmem’, ‘BRS Cora’, ‘BRS Violeta’ and ‘IAC 138–22 Máximo’ were assessed. These cultivars, from genetic improvement programs in Brazil, have good adaptation to subtropical and tropical climate conditions, and can be widely used by winegrowers aiming at adding value to the grape. All grapevines were grafted onto ‘IAC 766’ and ‘IAC 572’ rootstocks under tropical conditions in Brazil. The highest concentration of bioactive compounds was found in skins of ‘BRS Violeta’, followed by ‘IAC 138–22 Máximo’, both grafted onto ‘IAC 766’. There was a strong correlation between phenolic content and antioxidant properties, since antioxidant activity also decreased in the sequence: ‘BRS Violeta’ > ‘IAC 138–22 Máximo’ > ‘BRS Cora’ > ‘BRS Carmem’ > ‘Isabel Precoce’. Skin from hybrid grapes (‘BRS Violeta’, ‘IAC 138–22 Máximo’, ‘BRS Cora’ and ‘BRS Carmem’) grafted in both rootstocks contains higher levels of (poly)phenolic compounds and antioxidant activity than ‘Isabel Precoce’ (*V*. *labrusca*). Skin from ‘BRS Violeta’ grafted onto ’IAC 766’ stand out from the others due to their high content of bioactive compounds.

## Introduction

Brazilian grape juices are mainly produced from *Vitis labrusca* grapes, such as ‘Isabel’, ‘Bordô’ and ‘Concord’. These varieties are well suited to the temperate climate of southern Brazil and account for over 80% of national production. These grapes are destined to produce jams, jellies and table wines that are made from non-vinifera grapes, but this scenario has been changing [1, 2].

Regarding grape processing by-products, the development of new hybrid grape varieties that are adapted to warmer regions has enabled the extension of grape growing areas in the tropical regions of Brazil [3, 4]. Furthermore, new hybrids have been developed by the Brazilian Agricultural Research Corporation (Embrapa), such as ‘BRS Carmem’, ‘BRS Cora’ and ‘BRS Violeta’; and by the Agronomic Institute of Campinas (IAC), such as’ IAC 138–22 Máximo’. All of them are considered raw materials for grape juices and table wines making and knowledge of the grapes’ chemical composition is essential to assess the quality of the finished product.

In grapes, phenolic compounds are present predominantly in the peel and seeds. In grape skin there are mainly anthocyanins and resveratrol isomers, while seeds have a predominance of flavanols, such as (+)-catechin and (–)-epicatechin. In turn, phenolic acids are mainly found in the pulp [5, 6]. Studies show that phenolic compounds can act as antioxidants that scavenge free radicals [6–8] besides that, intake of polyphenol-rich foods may be associated with decreased risk of chronic diseases, e.g. cardiovascular disease, cancer and diabetes [9].

In addition to phenolic acids, grape pulp also concentrates primary metabolites, such as organic acids and sugars [10]. Glucose, fructose, and tartaric and malic acids are compounds that most contribute to grapes’ sweetness and acidity [11, 12]. These traits significantly influence their organoleptic quality and consequently affect the quality of juices and wines [13, 14]. Furthermore, studies have revealed that organic acids can also promote beneficial health effects. The addition of citric and malic acids to the diet has been shown to provide protective effects on the myocardium, acting on ischemic injuries [15].

Since grapes offer a wealth of health benefits, there is a need to consider their chemical composition, as it can be affected by several factors, such as soil composition, cultivation system, crop management practices, exposure of bunches to the sun and the incidence of pathogens [6, 16]. Furthermore, grape quality can also be strongly influenced by the rootstock. Studies show that rootstocks can significantly influence the bioactive content and antioxidant activity of different grape species [11, 17–19]. However, this influence depends on the specific affinity of the scion–rootstock interaction [20, 21].

In the southeastern region of Brazil, where a humid tropical climate (*Aw*) predominates, the cultivation of *V*. *vinifera*, which is mostly grown for fresh consumption, the ‘IAC 572’ rootstock is widely used, as it is known to be vigorous. The introduction of *Vitis labrusca* varieties and new hybrids destined for grape juice making requires further research to evaluate the interactions within scion–rootstock combinations. Previous studies have shown that ‘IAC 766’ rootstock, which is less vigorous than ‘IAC 572’, provides a higher yield [21, 22]. However, further studies are needed to characterize the phytochemical compounds in grapes. In this context, the current study aimed to evaluate the phytochemical profile of new Brazilian grape varieties grown on two Brazilian rootstocks in a region with a tropical climate in southeastern Brazil.

## Materials and methods

### Experimental location, grape varieties and growing conditions

Grapes were harvested from an experimental vineyard located in Votuporanga (20°20’ S and 49°58’ W, altitude 525 m), in the northwest region of the State of São Paulo, Brazil. According to the Köppen classification, the climate is Aw type, i.e., a tropical climate with a dry winter. An automatic meteorological station (Campbell Scientific^®^, Logan, UT, USA) installed in the experimental area recorded meteorological conditions during the study period. The mean temperature was 24.1°C, the minimum average was 16.6°C and the maximum average was 31.7°C. The average annual rainfall was 1495 mm, with a tendency for concentrated rainfall in the summer months. The soil was classified as ‘Argissolo Vermelho-Amarelo’ (equivalent to Ultisol, USDA soil taxonomy) according to previously published criteria [23].

‘Isabel Precoce’ (*V*. *labrusca* L.) and the hybrids ‘BRS Carmem’ (Muscat Belly A × H 65.9.14), ‘BRS Cora’ (Muscat Belly A × H. 65.9.14), ‘BRS Violeta’ (BRS Rubea × IAC 1398–21) and ‘IAC 138–22 Máximo’ (Seibel 11342 × Syrah), encoded as IP, CM, CR, VL and MX, respectively, were studied. The vines were grafted on ‘IAC 766 Campinas’ (106–8 Mgt × *V*. *caribaea*) and ‘IAC 572 Jales’ rootstocks [*V*. *caribaea* × 101–14 Mgt (*V*. *riparia* × *V*. *rupestris*)]. Thus, 10 scion–rootstock combinations were evaluated: IP-766, IP-572, CM-766, CM-572, CR-766, CR-572, VL-766, VL-572, MX-766 and MX-572.

The 3-year-old vines were trained on a unilateral cordon system (1 m above the soil) with vertical shoot positioning and spaced 2.0 × 1.1 m apart. Winter pruning was performed in August 2015 and the grapes were harvested in December 2015, when they reached the technological maturation stage [22]. Classical techniques were used to determine the pH, soluble solids and titratable acidity in grape samples. Values ranged from 3.18 to 3.29 for pH, 14.3 to 15.5°Brix for soluble solids (Brasil, 2002; IN 14, 2018) and from 0.66% to 1.14% tartaric acid for titratable acidity.

### Sample preparation and extraction

A sample of 200 berries was randomly collected from the basal, median and apical regions of bunches, divided into four replicates (4 plots, each plot with 50 berries) and fractionated into portions of skin and pulp. The seeds were discarded. The skin and pulp fractions were immediately frozen in liquid nitrogen and stored at −20°C until analysis.

For the determination of phenolic compounds and antioxidant activity, four plots (each plot with 50 berries) were used for the analyses. Skin and pulp fractions were extracted using > 99% (v/v) ethanol at a sample to solvent ratio of 1 : 2 w/v. The extracts were mixed and incubated for 1 h in a refrigerated shaker (20°C; Tecnal^®^, Brazil). After centrifugation at 4500 x *g* for 7 min, 1.5 mL of the supernatant was collected and the residual ethanol was evaporated by a vacuum centrifugal concentrator (miVac DUO concentratior, Genevac^®^, UK). Then, the extract was re-dissolved (1.5 mL final volume) in acidified water solution (1% phosphoric acid, v/v), filtered through a 0.45 μm membrane (Allcrom^®^, Brazil) and kept frozen at −80°C until analysis.

From a second sampling, only the pulp portions were used for extraction and analysis of organic acids and individual sugars. In these analyses, other 50 berries were used in each plot (4 plots). The pulp was mixed and diluted in 1 : 1 (v/v) ultrapure water. The extracts were filtered through 0.45 μm nylon membranes (Allcrom^®^, Brazil) and kept frozen at −80°C until analysis.

### Organic acids and sugars determined by HPLC

Organic acids (tartaric, malic, citric, lactic and acetic acids) and individual sugars (glucose and fructose) in pulp were determined using an Agilent 1260 Infinity LC HPLC system (Agilent Technologies, Santa Clara, CA, USA) coupled to a diode array detector (DAD, model G1315D) and refractive index detector (RID, model G1362A), according to the methodology described and validated by Coelho et al. [13]. Separation of the compounds was performed on a Hi-Plex H ion exchange column (300 × 7.7 mm, 8.0 μm internal particles) protected by a PL Hi-Plex H (5 × 3 mm) guard column (Agilent Technologies, Santa Clara, CA, USA). The phase was 0.004 mol L^−1^ H_2_SO_4_ in ultrapure water.

### Total phenolic compounds and total monomeric anthocyanins

The total phenolic compound content of the skin and pulp extracts was determined by the Folin–Ciocalteu spectrophotometric method [24]. The absorbance value at 765 nm was compared to a calibration curve and the results were expressed as milligrams of gallic acid equivalent (GAE) per kilogram of skin or pulp (mg kg^−1^). The total monomeric anthocyanin content was determined using the pH-differential method [25] and expressed as malvidin 3,5-diglucoside equivalents in mg L^−1^. Both analysis techniques were performed using an UV-Vis spectrophotometer (Instrutherm^®^ 2000A, Brazil).

### Profile of phenolic compounds

The profile of phenolic compounds was determined using a Waters Alliance e2695 liquid chromatograph equipped with a DAD (Waters, Milford, MA, USA) [26]. The column used was a Gemini-NX C18 (150 mm × 4.60 mm, with 3 μm internal particles) and the pre-column was a Gemini-NX C18 (4.0 mm × 3.0 mm), both manufactured by Phenomenex (USA).

### *In vitro* antioxidant activity

The *in vitro* antioxidant activity was determined using the 2,2’-azino-bis(3-ethylbenzothiazoline-6-sulfonic acid) (ABTS) [27] and 2,2-diphenyl-1-picrylhydrazyl (DPPH) radical scavenging methods [28]. For the analysis, the skin and pulp extract samples were diluted with ultrapure water until between 20% and 80% inhibition of the DPPH and ABTS radicals was obtained. The 1 mM DPPH and ABTS radical solutions were prepared in ethanol and diluted to an absorbance of 0.900 ± 0.050 (λ = 734 nm) and 0.700 ± 0.050 (λ = 517 nm), respectively. The absorbances were determined before and after the addition of grape juice using a UV-Vis 2000A spectrophotometer (Instrutherm^®^, São Paulo, SP, Brazil). In the DPPH method, absorbance was measured at times t = 0 and t = 30 min after addition of the sample. In the ABTS method, the absorbance was determined at times t = 0 and t = 6 min after addition of the samples. For both methods, the analytical standard Trolox was used to construct the calibration curves and the results were expressed as equivalents of Trolox per kilogram of grape skin or grape pulp (mM TEAC kg^−1^).

### Experimental design and statistical analysis

A completely randomized experimental design was used with ten treatments (scion/rootstock combinations) with four replicates. Data from skin and pulp analysis were submitted to one-way analysis of variance (ANOVA) and means were compared by Tukey test at 5% probability of error, using the statistical program SISVAR version 5.4 [29]. Principal component analysis (PCA) was applied to the 60 variables analyzed in grape skin and pulp, performed through XLSTAT software version 19.4 (Addinsoft, NY, USA).

## Results and discussion

### Organic acids and sugars

In grapes, organic acids and sugars are mainly accumulated in the pulp [10, 12]. Tartaric acid was the major compound (4.23 to 6.41 g L^−1^) in most red grapes, except in ‘IP-766’, which had the highest content of malic acid (7.77 g L^−1^) (Table 1). Therefore, both tartaric and malic acids corresponded to 92.0% and 98.0% of the organic acids quantified. Nevertheless, it has been already confirmed that those acids may account for more than 90.0% of the organic acids found in grapes [10]. The lowest organic acid content was observed in ‘CM-572’ (7.47 g L^−1^) and the presence of acetic and lactic acids was not detected in any grape berries, which is desirable, since these acids affect stability and characterize microbiological changes in beverages [14].

**Table 1:** Organic acids and sugars (g L^-1^) in grape pulps of *Vitis labrusca* and hybrid grown on rootstocks under tropical conditions in south-eastern Brazil.

Scion	‘Isabel Precoce’ (IP)				‘BRS Carmem’ (CM)				‘BRS Cora’ (CR)				‘BRS Violeta’ (VL)				‘IAC 138–22 Máximo’ (MX)			
Rootstock	IAC 766		IAC 572		IAC 766		IAC 572		IAC 766		IAC 572		IAC 766		IAC 572		IAC 766		IAC 572	
**Organic acids**																				
Tartaric	5.33	± 0.33 ^cd^	5.54	± 0.25 ^bc^	6.41	± 0.33 ^a^	4.28	± 0.38 ^e^	4.75	± 0.07 ^de^	5.03	± 0.17 ^cd^	4.23	± 0.27 ^e^	4.87	± 0.09 ^de^	6.19	± 0.33 ^ab^	5.33	± 0.33 ^cd^
Malic	7.77	± 0.47 ^a^	3.22	± 0.27 ^bc^	2.74	± 0.43 ^bc^	2.96	± 2.17 ^bc^	3.75	± 0.15 ^bc^	4.26	± 0.23 ^b^	3.37	± 0.17 ^bc^	2.74	± 0.23 ^bc^	3.53	± 0.04 ^bc^	2.30	± 0.07 ^c^
Citric	0.43	± 0.14 ^b^	0.45	± 0.07 ^b^	0.38	± 0.02 ^b^	0.23	± 0.01 ^c^	0.73	± 0.02 ^a^	0.72	± 0.05 ^a^	0.15	± 0.00 ^c^	0.15	± 0.00 ^c^	0.62	± 0.05 ^a^	0.46	± 0.02 ^b^
Acetic	ND		ND		ND		ND		ND		ND		ND		ND		ND		ND	
Lactic	ND		ND		ND		ND		ND		ND		ND		ND		ND		ND	
Σ Organic acids	13.54	± 3.62	9.22	± 2.47	9.52	± 2.76	7.47	± 2.00	9.23	± 2.24	10.01	± 2.45	7.76	± 2.08	7.76	± 2.19	10.34	± 2.73	8.09	± 2.28
**Sugars**																				
Glucose	79.95	± 0.28 ^bc^	74.27	± 0.24 ^d^	87.52	± 0.34 ^a^	84.20	± 0.41 ^ab^	74.70	± 0.30 ^cd^	73.06	± 1.73 ^d^	76.82	± 3.30 ^cd^	76.57	± 6.22 ^cd^	75.52	± 0.36 ^cd^	72.66	± 0.56 ^d^
Fructose	73.83	± 0.40 ^abc^	66.83	± 0.15 ^def^	78.20	± 0.25 ^a^	75.73	± 0.29 ^ab^	73.42	± 0.47 ^abc^	71.31	± 1.99 ^bcd^	70.19	± 2.99 ^cde^	70.28	± 5.35 ^cde^	65.92	± 0.67 ^ef^	64.24	± 0.18 ^f^
Σ sugars	153.78	± 4.33	141.09	± 5.26	165.72	± 6.59	159.93	± 5.99	148.12	± 0.90	144.37	± 1.23	147.01	± 4.69	146.85	± 4.45	141.45	± 6.79	136.89	± 5.96
Ratio Glu/Fruct		1.08	1.11			1.12	1.11			1.02	1.02			1.09	1.08			1.15	1.13	

Mean values are reported as the mean ± standard deviation (*n* = 4). Different letters within a row indicate a significant difference according to Tukey’s test (*p* < 0.05). ND–not detected. Σ–total compounds quantified by HPLC in each class. The 10 scion-rootstock combinations evaluated were named as: IP-766, IP-572, CM-766, CM-572, CR-766, CR-572, VL-766, VL-572, MX-766 and MX-572.

The highest content of glucose (85.86 g L^−1^) and fructose (77.24 g L^−1^) was observed in ‘BRS Carmem’. All grapes showed a glucose/fructose ratio ranging from 1.02 to 1.15, but also a slightly higher glucose-to-fructose-ratio was observed in ‘IAC 138–22 Máximo’, due to its lower fructose content. Notwithstanding that, these data are close to average (i.e. 1.0) for late-ripening grapes [12].

The content and composition of organic acids and soluble sugars determine the organoleptic quality and taste of grapes [10, 14], and these characteristics were influenced by the rootstocks. ‘BRS Carmem’ and ‘IAC 138–22 Máximo’ grafted on ‘IAC 766’ rootstock had the highest levels of tartaric and citric acids. In the same rootstock, ‘Isabel Precoce’ showed a high content of malic acid (7.77 g L^−1^), glucose (79.95 g L^−1^) and fructose (73.83 g L^−1^) (Table 1). Rootstocks can induce light uptake by grapevine canopies, directly affecting carbon assimilation and storage [17], factors that influence the metabolism of acids and sugars in plants. Variations in the content of malic and tartaric acids, as well as glucose and fructose, were also observed in ‘Syrah’ grapes grafted on ‘1103 Paulsen’ and ‘110 Richter’ rootstocks, as a function of their difference in vigor [30].

### Profile of phenolic compounds

**Fig 1:**
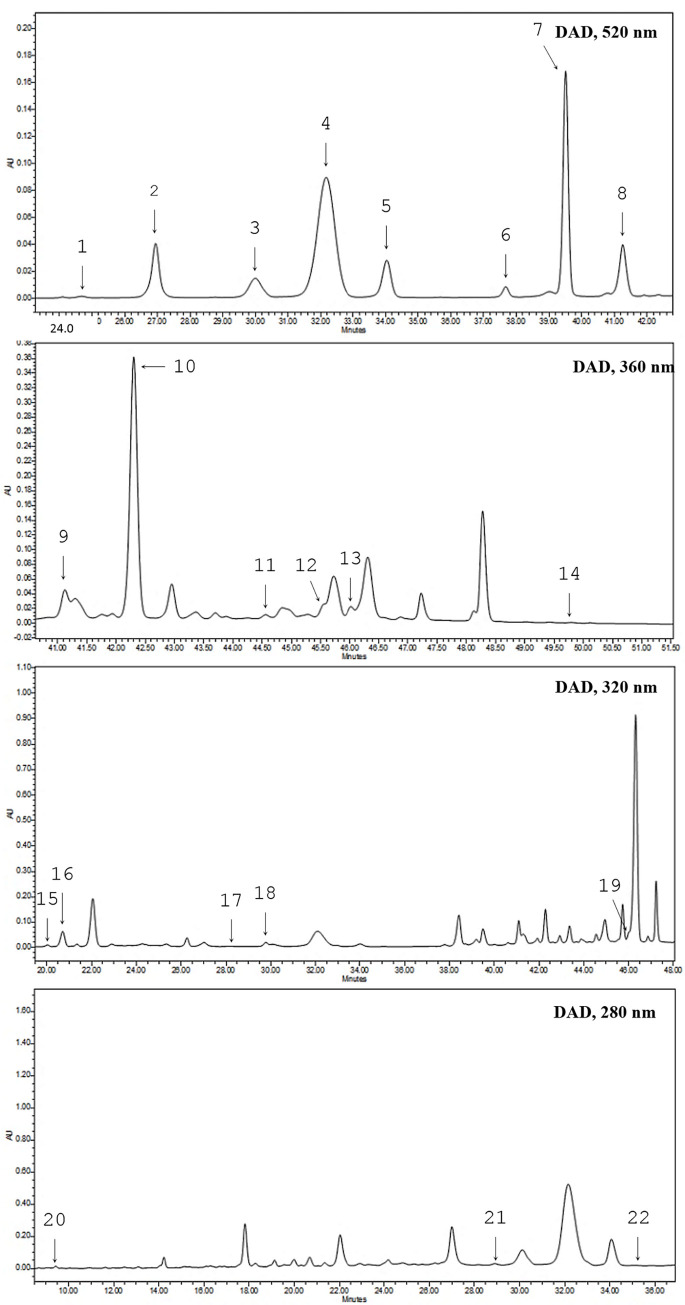
HPLC chromatograms from extracts of grape berry skin sample. Cyanidin 3,5-diglc [1], delphinidin 3-glc [2], cyanidin 3-glc [3], malvidin 3,5-diglc [4], pelargonidin 3-glc [5], peonidin 3-glc [6], malvidin 3-glc [7], petunidin 3-glc [8], rutin [9], isoquercetin [10], kaempferol [11], isorhamnetin [12], myricetin [13], quercetin [14], chlorogenic acid [15], caffeic acid [16], *ρ*-coumaric acid [17], cinnamic acid [18], *trans-*resveratrol [19], gallic acid [20], (–)-epigallocatechin gallate [21], (–)-epicatechin gallate [22].

Phenolic compounds were quantified separately in grape skin and pulp extracts from different scion/rootstock combinations. A typical chromatogram of grape skin extracts is shown in Fig 1.

#### Anthocyanin profile

The anthocyanin profile varied significantly according to the scion/rootstock (Table 2). The content in grape skins was higher than in pulps. In grape pulp, the anthocyanin content ranged from 0.28 to 11.59 mg kg^−1^. The levels of total anthocyanins detected in pulps and skins of ‘IAC 138–22 Máximo’ and ‘BRS Violeta’ grapes were the highest compared to the total content of anthocyanins in other grapes. Similar results have been described in ‘BRS Violeta’ and ‘Bordô’ (*V*. *labrusca*) grape pulps [3, 8]. However, the anthocyanin content reached 3025.5 mg kg^−1^ in VL-766 grape skin, the major anthocyanins in this variety being cyanidin-3,5-diglc (diglucoside), cyanidin-3-glc (glucoside) and delphinidin-3-glc. Such a result was significantly different from those for the other varieties, including VL-572. It is worth noting that the content of malvidin-3,5-diglc in VL-766 (805.3 mg kg^−1^) was higher than that in VL-572 (571.8 mg kg^−1^), evidencing the affinity of ‘BRS Violeta’ for ‘IAC 766’.

**Table 2:** Phenolic compounds (mg Kg^-1^) in grape skin and pulps of *Vitis labrusca* and hybrids grown on ‘IAC 766’ and ‘IAC 572’ rootstocks under tropical climate conditions in south-eastern Brazil.

Scion	Grape portion	‘Isabel Precoce’ (IP)				‘BRS Carmem’ (CM)				‘BRS Cora’ (CR)				‘BRS Violeta’ (VL)				‘IAC 138–22 Máximo’ (MX)			
Rootstock		IAC 766		IAC 572		IAC 766		IAC 572		IAC 766		IAC 572		IAC 766		IAC 572		IAC 766		IAC 572	
**Anthocyanins**																					
Malvidin 3,5-diglc	Skin	34.43	± 7.11 ^e^	39.89	± 6.55 ^e^	290.2	± 47.0 ^d^	324.3	± 22.6 ^d^	ND		ND		805.3	± 81.6 ^ab^	571.8	± 81.3 ^c^	878.2	± 49.7 ^a^	738.5	± 39.0 ^b^
	Pulp	ND		ND		0.70	± 0.24 ^c^	1.25	± 0.20 ^bc^	ND		ND		3.67	± 0.76 ^a^	1.44	± 0.53 ^bc^	3.01	± 0.71 ^ab^	3.66	± 1.74 ^a^
Malvidin 3-glc	Skin	181.7	± 44.9 ^b^	199.6	± 18.1 ^b^	8.24	± 3.28 ^c^	7.61	± 1.49 ^c^	ND		ND		116.7	± 50.5 ^b^	8.64	± 2.75 ^c^	376.3	± 65.3 ^a^	314.2	± 37.4 ^a^
	Pulp	0.21	± 0.05 ^b^	0.27	± 0.23 ^b^	0.05	± 0.02 ^b^	0.06	± 0.03 ^b^	ND		ND		0.15	± 0.09 ^b^	0.04	± 0.00 ^b^	0.73	± 0.27 ^a^	0.89	± 0.32 ^a^
Cyanidin 3,5-diglc	Skin	ND		ND		1.07	± 0.43 ^c^	0.83	± 0.25 ^c^	71.42	± 19.8 ^c^	71.14	± 8.99 ^c^	498.5	± 69.9 ^a^	391.7	± 62.6 ^b^	6.59	± 2.60 ^c^	7.69	± 1.21 ^c^
	Pulp	ND		ND		ND		ND		0.37	± 0.30 ^b^	0.17	± 0.09 ^b^	2.63	± 0.70 ^a^	1.78	± 0.65 ^a^	ND		ND	
Cyanidin 3-glc	Skin	15.12	± 3.66 ^d^	11.49	± 2.98 ^d^	25.35	± 7.06 ^d^	24.80	± 1.55 ^d^	32.00	± 8.47 ^cd^	35.43	± 4.29 ^cd^	255.6	± 35.5 ^a^	172.4	± 31.8 ^b^	67.51	± 20.8 ^c^	72.39	± 10.2 ^c^
	Pulp	ND		ND		0.44	± 0.11 ^c^	0.56	± 0.08 ^c^	0.22	± 0.18 ^c^	0.13	± 0.08 ^c^	3.37	± 0.73 ^a^	1.72	± 0.60 ^b^	0.51	± 0.11 ^c^	0.61	± 0.27 ^c^
Delphinidin 3-glc	Skin	19.28	± 4.95 ^e^	16.39	± 1.91 ^e^	11.06	± 3.66 ^e^	9.96	± 1.71 ^e^	252.5	± 56.2 ^c^	278.5	± 35.6 ^c^	1127	± 53.6 ^a^	888.0	± 72.5 ^b^	117.0	± 53.1 ^d^	101.8	± 23.9 ^de^
	Pulp	ND		ND		ND		ND		0.13	± 0.08 ^c^	0.06	± 0.05 ^c^	1.31	± 0.32 ^a^	0.63	± 0.22 ^b^	0.06	± 0.03 ^c^	0.07	± 0.03 ^c^
Peonidin 3-glc	Skin	38.83	± 7.96 ^a^	36.19	± 6.08 ^a^	1.62	± 0.73 ^c^	1.52	± 0.26 ^c^	2.72	± 0.69 ^c^	2.59	± 0.31 ^c^	12.82	± 1.84 ^b^	7.15	± 1.17 ^bc^	12.71	± 5.42 ^b^	12.87	± 3.17 ^b^
	Pulp	0.07	± 0.04 ^a^	0.09	± 0.06 ^a^	0.08	± 0.03 ^a^	0.11	± 0.02 ^a^	ND		ND		0.15	± 0.06 ^a^	0.10	± 0.05 ^a^	0.05	± 0.02 ^a^	0.07	± 0.04 ^a^
Pelargonidin 3-glc	Skin	32.96	± 8.48 ^cd^	32.42	± 3.23 ^cd^	5.45	± 2.54 ^d^	4.41	± 1.20 ^d^	57.58	± 9.69 ^c^	54.60	± 7.19 ^c^	184.8	± 6.15 ^a^	154.4	± 2.99 ^ab^	122.8	± 50.8 ^bc^	109.4	± 22.5 ^c^
	Pulp	ND		ND		ND		ND		ND		ND		0.23	± 0.06 ^a^	0.10	± 0.02 ^b^	0.07	± 0.03 ^b^	0.09	± 0.05 ^b^
Petunidin 3-glc	Skin	ND		ND		ND		ND		ND		ND		24.27	± 4.97 ^b^	11.02	± 1.29 ^b^	58.98	± 7.15 ^a^	55.21	± 9.85 ^a^
	Pulp	ND		ND		ND		ND		ND		ND		0.08	± 0.00 ^bc^	0.06	± 0.02 ^c^	0.13	± 0.02 ^ab^	0.14	± 0.04 ^a^
Σ Antocyanins	Skin	322.37	± 59.07	336.02	± 65.56	342.9	± 100.2	373.4	± 112.5	416.21	± 85.81	442.29	± 94.43	3025.5	± 403.2	2205.1	± 319.6	1640.0	± 296.1	1412.0	± 246.6
	Pulp	0.28	± 0.07	0.36	± 0.10	1.37	± 0.26	2.12	± 0.44	0.72	± 0.14	0.36	± 0.07	11.59	± 1.55	5.86	± 0.79	4.56	± 1.02	5.53	± 1.24
**Flavonols**																					
Quercetin	Skin	ND		ND		ND		ND		ND		ND		ND		ND		0.10	± 0.00 ^a^	0.10	± 0.00 ^a^
	Pulp	ND		ND		ND		ND		ND		ND		ND		ND		ND		ND	
Isoquercetin	Skin	29.69	± 4.06 ^b^	23.13	± 8.64 ^bc^	4.54	± 0.41 ^d^	7.35	± 2.87 ^d^	24.02	± 8.41 ^bc^	29.39	± 3.26 ^b^	33.18	± 8.61 ^ab^	12.04	± 1.57 ^cd^	34.16	± 4.72 ^ab^	45.49	± 6,63 ^a^
	Pulp	ND		ND		ND		ND		ND		ND		0.23	± 0.07 ^a^	0.13	± 0.05 ^a^	ND		ND	
Rutin	Skin	1.22	± 0.09 ^b^	0.64	± 0.55 ^b^	0.77	± 0.22 ^b^	0.80	± 0.16 ^b^	4.59	± 0.34 ^a^	4.57	± 1.11 ^a^	1.24	± 0.45 ^b^	1.31	± 0.39 ^b^	4.55	± 0.58 ^a^	5.35	± 0,82 ^a^
	Pulp	ND		ND		ND		ND		ND		ND		ND		ND		ND		ND	
Kaempferol	Skin	2.23	± 0.44 ^bc^	1.31	± 1.15^bcd^	1.55	± 0.39^bcd^	2.39	± 0.72 ^b^	7.38	± 1.56 ^a^	7.23	± 0.95 ^a^	0.52	± 0.09 ^cd^	0.35	± 0.06 ^d^	1.28	± 0.12^bcd^	1.65	± 0,25^bcd^
	Pulp	ND		ND		ND		ND		ND		ND		0.14	± 0.05 ^a^	0.09	± 0.06 ^a^	ND		ND	
Myricetin	Skin	1.79	± 0.25 ^bc^	1.24	± 0.6 ^cdef^	0.57	± 0.22 ^efg^	0.75	± 0.2 ^defg^	0.32	± 0.05 ^g^	0.42	± 0.09 ^fg^	4.28	± 0.41 ^a^	2.58	± 0.40 ^b^	1.38	± 0.51^cde^	1.50	± 0,25 ^cd^
	Pulp	ND		ND		0.04	± 0.00 ^b^	0.07	± 0.02 ^a^	ND		ND		0.04	± 0.00 ^b^	0.04	± 0.00 ^b^	0.04	± 0.00 ^b^	0.04	± 0.00 ^b^
Isorhamnetin	Skin	0.40	± 0.11 ^d^	0.32	± 0.05 ^d^	0.40	± 0.20 ^d^	0.35	± 0.06 ^d^	1.65	± 0.26 ^cd^	1.57	± 0.30 ^cd^	22.68	± 4.09 ^a^	16.51	± 2.41 ^b^	4.11	± 0.43 ^c^	5.00	± 0,49 ^c^
	Pulp	ND		ND		0.06	± 0.02 ^a^	0.07	± 0.02 ^a^	ND		ND		0.12	± 0.06 ^a^	0.07	± 0.04 ^a^	ND		ND	
Σ Flavonols	Skin	35.33	± 11.69	26.64	± 9.17	7.83	± 1.67	11.64	± 2.77	37.96	± 9.11	43.19	± 11.22	61.89	± 14.12	32.80	± 7.03	45.58	± 13.13	59.09	± 17,58
	Pulp	0.00	± 0.00	0.00	± 0.00	0.10	± 0.03	0.14	± 0.04	0.00	± 0.00	0.00	± 0.00	0.53	± 0.09	0.33	± 0.05	0.04	± 0.02	0.04	± 0,02
**Stilbene**																					
*trans*-resveratrol	Skin	0.52	± 0.09 ^d^	0.60	± 0.08 ^d^	1.67	± 0.37 ^bc^	1.57	± 0.18 ^c^	0.52	± 0.05 ^d^	0.45	± 0.10 ^d^	3.89	± 0.57 ^a^	2.36	± 0.49 ^b^	1.43	± 0.20 ^c^	1.40	± 0.12 ^c^
	Pulp	0.08	± 0.00 ^c^	0.08	± 0.00 ^c^	0.12	± 0.00 ^b^	0.12	± 0.00 ^b^	0.08	± 0.00 ^c^	0.08	± 0.00 ^c^	0.17	± 0.02 ^a^	0.12	± 0.03 ^b^	0.11	± 0.02 ^bc^	0.13	± 0.02 ^b^
**Phenolic acids**																					
Gallic acid	Skin	2.76	± 0.82 ^b^	2.03	± 0.57 ^b^	2.67	± 0.32 ^b^	3.29	± 1.50 ^ab^	3.49	± 0.42 ^ab^	3.07	± 0.68 ^ab^	4.63	± 0.41 ^a^	3.01	± 0.55 ^ab^	2.61	± 0.50 ^b^	2.87	± 0.69 ^b^
	Pulp	3.10	± 0.20 ^a^	1.98	± 0.48 ^bc^	1.60	± 0.21^cde^	1.92	± 0.23^bcd^	2.30	± 0.21 ^b^	1.64	± 0.4 ^bcde^	1.14	± 0.17 ^e^	1.30	± 0.29 ^de^	1.41	± 0.14^cde^	1.23	± 0.19 ^e^
Caffeic acid	Skin	14.19	± 5.55 ^ab^	15.69	± 2.32 ^a^	1.62	± 0.43 ^d^	1.40	± 0.14 ^d^	8.16	± 0.50 ^c^	7.35	± 0.96 ^c^	9.40	± 0.86 ^bc^	10.50	± 3.95^abc^	7.08	± 1.17 ^cd^	7.98	± 0.31 ^c^
	Pulp	0.49	± 0.10^abc^	0.42	± 0.18^bcd^	0.11	± 0.07 ^de^	0.21	± 0.09^cde^	0.42	± 0.15^bcd^	0.31	± 0.09^cde^	0.77	± 0.19 ^a^	0.70	± 0.21 ^ab^	0.08	± 0.03 ^e^	0.14	± 0.02 ^de^
Cinnamic acid	Skin	0.65	± 0.26 ^b^	0.82	± 0.23 ^b^	0.65	± 0.18 ^b^	0.50	± 0.29 ^b^	0.50	± 0.08 ^b^	0.52	± 0.10 ^b^	11.52	± 1.74 ^a^	12.11	± 2.34 ^a^	0.83	± 0.09 ^b^	0.75	± 0.13 ^b^
	Pulp	ND		ND		ND		ND		ND		ND		ND		ND		ND		ND	
Chlorogenic acid	Skin	2.03	± 0.63^bcd^	1.99	± 0.25^bcd^	0.12	± 0.05 ^d^	0.18	± 0.05 ^d^	0.60	± 0.00 ^cd^	0.60	± 0.08 ^cd^	8.76	± 3.61 ^a^	4.51	± 0.57 ^b^	1.41	± 0.54 ^cd^	3.32	± 0.68 ^bc^
	Pulp	0.13	± 0.04 ^cd^	0.12	± 0.03 ^cd^	0.04	± 0.00 ^d^	0.04	± 0.00 ^d^	0.20	± 0.06 ^bc^	0.25	± 0.06 ^b^	0.04	± 0.00 ^d^	0.04	± 0.00 ^d^	0.42	± 0.05 ^a^	0.50	± 0.05 ^a^
*ρ*-Coumaric acid	Skin	0.35	± 0.10 ^c^	0.40	± 0.08 ^c^		ND		ND	0.32	± 0.05 ^c^	0.40	± 0.08 ^c^	1.01	± 0.10 ^a^	0.68	± 0.18 ^b^	0.23	± 0.05 ^c^	0.23	± 0.05 ^c^
	Pulp	0.04	± 0.00 ^b^	0.04	± 0.00 ^b^	0.04	± 0.00 ^b^	0.04	± 0.00 ^b^	0.07	± 0.02 ^ab^	0.06	± 0.02 ^ab^	0.06	± 0.02 ^ab^	0.05	± 0.02 ^ab^	0.08	± 0.00 ^a^	0.06	± 0.02 ^ab^
Siringic acid	Skin	ND		ND		ND		ND		ND		ND		ND		ND		ND		ND	
	Pulp	ND		ND		ND		ND		ND		ND		ND		ND		ND		ND	
Σ Phenolic acids	Skin	19.97	± 5.42	20.93	± 6.04	5.07	± 1.09	5.37	± 1.29	13.07	± 3.19	11.94	± 2.85	35.31	± 4.74	30.81	± 5.07	12.15	± 2.65	15.15	± 3.01
	Pulp	3.76	± 1.23	2.56	± 0.78	1.79	± 0.64	2.21	± 0.76	2.98	± 0.90	2.26	± 0.63	2.01	± 0.50	2.09	± 0.54	1.98	± 0.55	1.93	± 0.48
**Flavanols**																					
(–)-Epic. gallate	Skin	1.66	± 0.40 ^d^	1.36	± 0.16 ^d^	1.87	± 0.26 ^cd^	2.15	± 0.48 ^cd^	2.84	± 0.43 ^bc^	2.79	± 0.46 ^bc^	4.38	± 0.43 ^a^	3.29	± 1.02 ^ab^	1.21	± 0.16 ^d^	1.58	± 0.14 ^d^
	Pulp	0.26	± 0.05 ^ab^	0.29	± 0.16 ^ab^	0.27	± 0.04 ^ab^	0.30	± 0.02 ^ab^	0.13	± 0.02 ^bc^	0.12	± 0.03 ^b^	0.46	± 0.21 ^a^	0.34	± 0.09 ^ab^	0.24	± 0.06 ^ab^	0.26	± 0.10 ^ab^
(–)-Epig. gallate	Skin	0.89	± 0.56^cde^	0.62	± 0.26 ^de^	2.52	± 0.68 ^ab^	2.90	± 0.70 ^a^	0.23	± 0.10 ^e^	0.30	± 0.12 ^e^	1.82	± 0.55 ^bc^	1.41	± 0.44 ^cd^	1.63	± 0.32^bcd^	1.93	± 0.22^abc^
	Pulp	0.24	± 0.03 ^b^	0.24	± 0.07 ^b^	0.45	± 0.09 ^a^	0.48	± 0.06 ^a^	0.15	± 0.08 ^b^	0.14	± 0.04 ^bc^	0.11	± 0.04 ^c^	0.09	± 0.04 ^c^	0.14	± 0.02 ^bc^	0.16	± 0.00 ^bc^
Σ Flavanols	Skin	2.56	± 0.55	1.98	± 0.53	4.39	± 0.46	5.05	± 0.53	3.08	± 1.85	3.09	± 1.76	6.19	± 1.81	4.69	± 1.33	2.84	± 0.30	3.50	± 0.25
	Pulp	0.50	± 0.01	0.53	± 0.04	0.72	± 0.13	0.77	± 0.13	0.28	± 0.01	0.26	± 0.01	0.57	± 0.25	0.43	± 0.18	0.38	± 0.07	0.42	± 0.07
**TMA** ^**1**^ **(g Kg**^**-1**^**)**	Skin	0.51	± 0.03 ^e^	0.53	± 0.02 ^e^	1.58	± 0.06 ^d^	1.72	± 0.06 ^d^	1.69	± 0.21 ^d^	1.77	± 0.14 ^d^	9.49	± 0.41 ^a^	8.55	± 0.36 ^b^	3.23	± 0.18 ^c^	2.85	± 0.14 ^c^
	Pulp	NA		NA		NA		NA		NA		NA		NA		NA		NA		NA	
**TPC** ^**2**^ **(g Kg**^**-1**^**)**	Skin	0.94	± 0.09 ^e^	1.09	± 0.12 ^e^	2.54	± 0.24 ^d^	2.54	± 0.15 ^d^	2.54	± 0.32 ^d^	2.70	± 0.26 ^d^	11.05	± 0.56 ^a^	9.25	± 0.47 ^b^	5.09	± 0.92 ^c^	5.45	± 0.25 ^c^
	Pulp	0.21	± 0.04 ^bc^	0.19	± 0.04 ^bc^	0.16	± 0.02 ^c^	0.18	± 0.01 ^c^	0.21	± 0.03 ^bc^	0.22	± 0.07 ^bc^	0.38	± 0.07 ^a^	0.29	± 0.02 ^b^	0.13	± 0.01 ^c^	0.17	± 0.03 ^c^

Mean values are reported as the mean ± standard deviation (*n* = 4). Different letters within a row indicate a significant difference according to Tukey’s test (*p* < 0.05). The 10 scion-rootstock combinations evaluated were named as: IP-766, IP-572, CM-766, CM-572, CR-766, CR-572, VL-766, VL-572, MX-766 and MX-572.

Abbreviations: glc, glucoside; diglc, diglucoside; Epic, (–)-Epicatechin gallate; Epig, (–)-Epigallocatechin gallate; ND, not detected; NA–not analyzed; Σ, total compounds quantified by HPLC in each phenolic class.

^1^ TMA, total monomeric anthocyanins quantified by the technique of difference in pH and expressed as equivalent to malvidin 3,5-diglucoside.

^2^ TPC, total phenolic compounds measured with Folin-Ciocalteau reagent and expressed as mg Kg^−1^ equivalent to gallic acid.

Therefore, rootstocks influenced on the anthocyanin content, especially in ‘BRS Violeta’, in agreement with previous studies that assessed this variety on ‘IAC 766’ and ‘106–8 Mgt’ rootstocks [31]. Research analyzing the effect of rootstock on hybrid grapes for juice and wine making is scarce, but our results are similar to those from studies on other species that also reported the influence of rootstocks on grape anthocyanin content, i.e. ‘Red Alexandria’ (*V*. *vinifera*) [18] and ‘Greco Nero n.’ (*V*. *vinifera*) [32].

According to Xi et al. [16], in grapes the anthocyanins are synthesized via the flavonoid biosynthetic pathway. However, it is not yet well understood how the rootstock affects the biosynthesis and the content of these compounds. The expression of flavonoid biosynthesis-related genes varies depending on the rootstock [33]. In our study, we also observed variations in the anthocyanin profile according to the rootstock. High concentrations of anthocyanins were found in MX-766 and MX-572 (1640 and 1412 mg kg^−1^) grape skin and more than 50% was represented by malvidin-3,5-diglc, at 878.2 and 738.5 mg kg^−1^, respectively. It is worth remembering that malvidin-3,5-diglc was detected at high levels in VL-766 and VL-572. In non-vinifera grape varieties, some studies have reported a large amount of anthocyanins derived from the 3,5-diglucoside group, mainly malvidin, considered markers in these grapes [3, 34].

The main anthocyanin found in ‘Isabel Precoce’ grape skin was malvidin-3-glc (190.6 mg kg^−1^), although it is a *V*. *labrusca* variety (Table 2). This result corroborates those of previous studies on grape juices elaborated from this variety, in which the content of malvidin-3-glc was higher than that of malvidin-3,5-diglc [26, 35]. Similar results have also been obtained for grape juices and wines made from ‘Isabel’ (*V*. *labrusca*), which is the most used variety for juice making in Brazil and from which ‘Isabel Precoce’ originated through a spontaneous somatic mutation [2]. The anthocyanin profile of each variety is commonly linked to its genetic inheritance, although it may be influenced by environmental conditions [3].

VL-766 presented a higher concentration of total monomeric anthocyanins (TMA) than VL-572, i.e. 9.49 and 8.55 g kg^−1^ of grape skin, respectively. The TMA content was 17 times higher in ‘BRS Violeta’ than in ‘Isabel Precoce’ (0.52 mg kg^−1^). Other studies showed low levels of total anthocyanins in grape juices and wines from ‘Isabel Precoce’ and ‘Isabel’ [1, 26]. Nevertheless, high levels of these compounds have already been observed in ‘BRS Violeta’ [31, 34]. Thus, ‘BRS Violeta’ becomes an important source of anthocyanins, capable of producing intense color expression in grape juices and young red wines.

#### Flavonols and *trans*-resveratrol

The total amount of flavonols was low in ‘BRS Carmem’, ‘BRS Violeta’ and ‘IAC 138–22 Máximo’ grape pulps, as values ranged from 0.04 to 0.53 mg kg^−1^; none were detected in ‘Isabel Precoce’ or ‘BRS Cora’ (Table 2). A low flavonol content was also found in ‘Bordô’ grape pulp (1.44 μmol kg^−1^), about 100 times less than in grape skin [8]. The flavonol content ranged from 7.83 to 61.89 mg kg^−1^ in grape skins and isoquercetin was the major phenolic compound in all samples of grapes, being responsible for more than 50% of the total flavonol content in most samples. The highest isoquercetin level was found in MX-572, MX-766 and VL-766.

VL-766 skin and pulp showed the highest levels of *trans*-resveratrol, i.e., 3.89 and 0.17 mg kg^−1^, respectively. ‘BRS Carmem’ and ‘IAC 138–22 Máximo’ had similar values, with an average of 1.52 mg kg^−1^. The same occurred among the ‘Isabel Precoce’ and ‘BRS Cora’ grapes, which averaged 0.52 mg kg^−1^. This value is similar to those reported by Burin et al. [1] in ‘Isabel’, ‘Concord’ and ‘Bordô’, i.e., 0.35, 0.64 and 0.86 mg kg^−1^, respectively. In ‘Bordô’ skin, the *trans*-resveratrol content may reach 6.27 mg kg^−1^, while its presence was not detected in pulp [8]. According to the authors, grapes with a *trans*-resveratrol content above 2.37 mg kg^−1^ are considered high resveratrol producers, as detected in VL-766 skin (Table 2).

#### Phenolic acids and flavanols

The phenolic acid content in grape skin was higher than in pulps and ranged from 5.07 to 35.31 mg kg^−1^. This result corroborates those of the studies conducted by Rebello et al. [34] and Lago-Vanzela et al. [8], who also observed a greater distribution of phenolic acids, especially hydroxycinnamic acids, in skins than in pulps of ‘BRS Violeta’ and ‘Bordô’ grapes, respectively. The highest levels of phenolic acids were obtained in ‘BRS Violeta’ and ‘Isabel Precoce’, with an average of 33.06 and 20.45 mg kg^−1^, respectively, followed by ‘IAC 138–22 Máximo’, ‘BRS Cora’ and ‘BRS Carmem’ (13.65, 12.51 and 5.22 mg kg^−1^, respectively). Phenolic acid was also observed, in ‘BRS Violeta’ > ‘Isabel Precoce’ > ‘BRS Cora’ grape juices from the São Francisco Valley (Northeast, Brazil) [26]. Phenolic acids were genotype-dependent, as were the other phenolic compounds. In grape skin, the major phenolic acid was gallic acid in ‘BRS Carmem’, cinnamic acid in ‘BRS Violeta’ and caffeic acid in ‘Isabel Precoce’, ‘BRS Cora’ and ‘IAC 138–22 Máximo’. In all grape pulps, gallic acid was the most abundant phenolic acid. Gallic acid is described as one of the most important phenolic compounds in grapes, as it is the precursor of all hydrolyzable and condensed tannins, that is, compounds of sensory interest [36].

The flavanol content was similar between cultivars, values ranging from 0.26 to 0.72 mg kg^−1^ in grape pulps and from 1.98 to 6.19 mg kg^−1^ in grape skins (Table 2). The latter grape fraction presented a high epicatechin gallate content in ‘BRS Violeta’, while the highest epigallocatechin gallate content was found in ‘BRS Carmem’. There was no effect of the rootstocks in each grape variety. Flavanols are tannins that are mainly located in grape skin and seeds [19], which explains the low values found in pulp. The amount of flavonols in ‘Greco Nero n.’ has been shown to be little influenced by rootstock, suggesting a strong genotypic influence of the scion on the accumulation of these compounds in grape skin [32].

### Total bioactive content and antioxidant activity in vitro

The total phenolic compounds (TPC) in grape skin (0.94 to 11.05 mg kg^−1^) was higher than in grape pulp (0.13 to 0.38 mg kg^−1^) (Table 2). In both, the highest concentration of these compounds was obtained in ‘BRS Violeta’; therefore, there was influence of the rootstocks on this variety. VL-766 had a higher TPC than VL-572, i.e., 11.05 and 9.25 mg kg^−1^ in grape skin, respectively. IAC 138–22 ‘Máximo’ showed a high TPC in grape skin regardless of the rootstock. Silva et al. [31] found no effect of ‘IAC 766’ and ‘106–8 Mgt’ rootstocks on TPC content of ‘BRS Violeta’ and ‘IAC 138–22 Máximo’, with values of 7.18 and 6.65 mg kg^−1^, respectively. The authors found the highest phenolic content in these varieties in relation to other vinifera and non-vinifera grape varieties.

**Fig 2:**
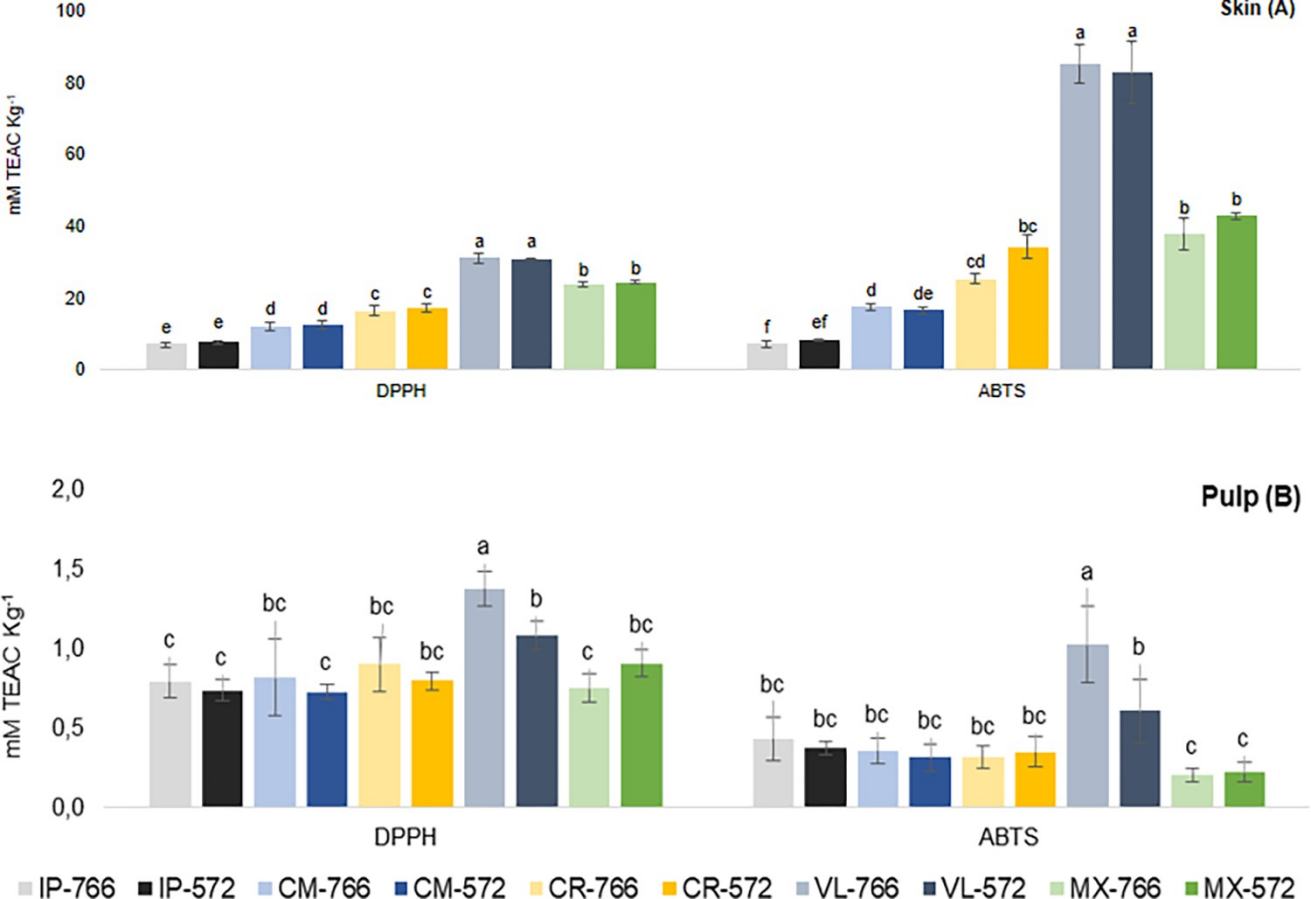
*In vitro* antioxidant activity of grape skin and pulp extracts of *Vitis labrusca* and hybrids. Vertical bars represent the mean value ± standard deviation (*n* = 4). Different letters within each method analyzed (DPPH and ABTS) indicate a significant difference according to Tukey’s test (*p* < 0.05). Samples: ‘Isabel Precoce’ [IP], ‘BRS Carmem’ [CM], ‘BRS Cora’ [CR], ‘BRS Violeta’ [VL], ‘IAC 138–22 Máximo’ [MX], ‘IAC 766’ rootstock [766], ‘IAC 572’ rootstock [572].

Grape skin had predictably higher antioxidant activity (AOX) than pulp, due to the higher content of phenolic compounds (Fig 2). In grape pulp, AOX ranged from 0.73 to 1.37 mM TEAC kg^−1^ for the DPPH method, and from 0.20 to 1.02 mM TEAC kg^−1^ for the ABTS method (Fig 2B). For both methods, high values were found in VL-766, differing significantly from VL-572. The effect of rootstock on the AOX of ‘BRS Violeta’ is related to the high phenolic content provided by ‘IAC 766’ rootstock, indicating the influence of rootstock on the phenolic compound content, as observed by Cheng et al. [18] in ‘Red Alexandria’ grape pulp.

Although the results for phenolic compounds showed an effect of rootstock on grape skins, mainly on ‘BRS Violeta’, there was no influence of ‘IAC 766’ and ‘IAC 572’ rootstock on the AOX of grape skin, which ranged from 7.20 to 30.79 mM TEAC kg^−1^ for the DPPH method and from 7.33 to 85.05 mM TEAC kg^−1^ for the ABTS method. In grape skins, the highest AOX (DPPH and ABTS) was observed in ‘BRS Violeta’ (30.9 and 83.9 mM TEAC kg^−1^), followed by IAC 138–22 ‘Máximo’ (24.1 and 40.4 mM TEAC kg^−1^) (Fig 2A). The lowest AOX was found in ‘Isabel Precoce’, 7.4 and 7.8 mM TEAC kg^−1^ using the DPPH and ABTS methods, respectively. The variation between grape varieties is related to the phenolic content, indicating that grapes rich in phenolic compounds also have higher AOX. Other studies have demonstrate these results not only in grapes, but in juices and wines [37, 38].

Significant correlations were found for DPPH and cyanidin 3,5-dglc (r = 0.76 and 0.95, skin and pulp, respectively), cyanidin 3-glc (r = 0.87 and 0.94), delphinidin 3-glc (r = 0.80 and 0.97) and pelargonidin 3-glc (r = 0.94 and 0.90) in skin and pulp, respectively, in addition to malvidin 3,5-diglc (r = 0, 79), isorhamnetin (r = 0.85) and trans-resveratrol (r = 0.73) in the skins and isoquercetin (r = 0.95) and kaempferol (r = 0.94) in pulp. When the ABTS method was applied, the phenolic compounds that most contributed to the antioxidant activity of the grapes were cyanidin 3,5-dglc (r = 0.91 and 0.93), cyanidin 3-glc (r = 0.95 and 0 .89), delphinidin 3-glc (r = 0.93 and 0.94) and isorhamnetin (r = 0.95 and 0.78) in skin and pulp, respectively, in addition to pelargonidin (r = 0.92), cinnamic acid (r = 0.91), chlorogenic acid (r = 0.81), ρ-coumaric acid (r = 0.77) and trans-resveratrol (r = 0.80) in the skin and isoquercetin (r = 0.95), kaempferol (r = 0.94) and caffeic acid (r = 0.85) in the pulps. Similarly, Rockenbach et al. [39] also observed significant positive correlations between phenolic compounds and antioxidant activity by the DPPH and FRAP methods in skin of *V*. *vinifera* and *V*. *labrusca* grapes. Significant correlations were also obtained between the antioxidant activity by the DPPH and ABTS methods and the total monomeric anthocyanins (r = 0.90 and 0.96, respectively), as well as the total phenolic compounds in the skin (r = 0.95 and 0 .98, respectively) and pulp (r = 0.91 and 0.96, respectively).

### Principal component analysis

**Fig 3:**
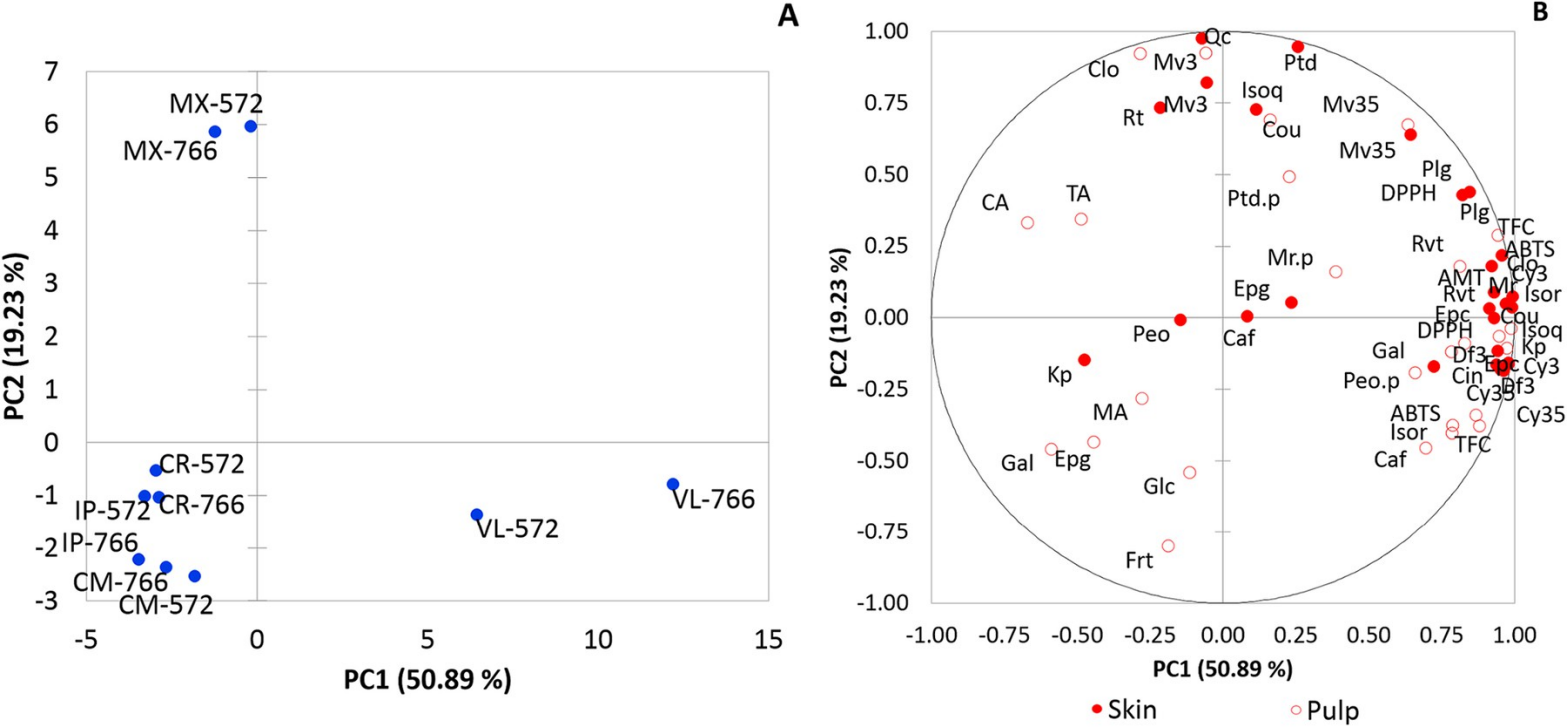
Principal component analysis in grape berry skin and pulps samples of *Vitis labrusca* and hybrids. CP1/CP2 scores (A) and loadings plot (B) explaining 70.12% of the total variation. Samples: ‘Isabel Precoce’ [IP], ‘BRS Carmem’ [CM], ‘BRS Cora’ [CR], ‘BRS Violeta’ [VL], ‘IAC 138–22 Máximo’ [MX], ‘IAC 766’ rootstock [766], ‘IAC 572’ rootstock [572]. Trait abbreviations: tartaric acid [TA], malic acid [MA], citric acid [CA], glucose [Glc], Frt [Frt], cyanidin 3,5-diglc [Cy35], delphinidin 3-glc [Df3], cyanidin 3-glc [Cy3], malvidin 3,5-diglc [Mv35], pelargonidin 3-glc [Plg], peonidin 3-glc [Peo], malvidin 3-glc [Mv3], petunidin 3-glc [Ptd], rutin [Rt], isoquercetin [Isoq], kaempferol [Kp], isorhamnetin [Isor], myricetin [Mr], quercetin [Qc], chlorogenic acid [Clo], caffeic acid [Caf], *ρ*-coumaric acid [Cou], cinnamic acid [Cin], *trans-*resveratrol [Rvt], gallic acid [Gal], (–)-epigallocatechin gallate [Epg], (–)-epicatechin gallate [Epc], total monomeric anthocyanins [TMA], total phenolic compounds [TFC], antioxidant activity by DPPH method [DPPH], antioxidant activity by ABTS method [ABTS].

Aiming at a descriptive model for grouping organic acids, sugars, phenolic compounds and AOX with scion/rootstock combinations, the results were compared using PCA. PC1 and PC2 explained 70.12% of the data variance (Fig 3). VL-766 and VL-572 grapes were grouped in PC1+ and PC2− (Fig 3A) and this result may be attributed to the phenolic compounds, mainly cyanidin-3,5-diglc, delfinidin-3-glc, cyanidin-3-glc, pelargonidin-3-glc, isorhamnetin, resveratrol and (−)-epicatechin gallate, besides TPC and AOX (DPPH and ABTS), related to grape skin and pulp (Table 2 and Fig 3B). In addition, other compounds such as myricetin, chlorogenic acid, ρ-coumaric acid, cinnamic acid, gallic acid and TMA found in grape skins, and isoquercetin and kaempferol from grape pulps contributed to the grouping in PC1+. It is worth mentioning that despite both being grouped in PC1+ and PC2−, the highest levels of these compounds were detected in VL-766, demonstrating the possible influence of ‘IAC 766’ rootstock on the antioxidant composition of grapes (Fig 3B and Table 2).

MX-766 and MX-572 grapes were grouped in PC1+ and PC2+ (Fig 3A) and this result is due to content of malvidin-3,5-diglc, malvidin-3-glc and petunidin-3-glc in both skin and pulp, isoquercetin, quercetin and rutin in grape skins, and malvidin-3-glc and chlorogenic acid in grape pulps (Table 2). PC2 scores and loadings suggest a higher concentration of these compounds in ‘IAC 138–22 Máximo’.

‘Isabel Precoce’, ‘BRS Cora’ and ‘BRS Carmem’ were grouped in PC1− and PC2− (Fig 3A), regardless of the rootstock. In ‘BRS Carmem’ pulp the highest levels of glucose and fructose were observed (Fig 3B), results that may have contributed to the grouping of these grapes in PC1− and PC2−.

## Conclusions

In this study, there were differences in the phytochemical profile of grapes grown on different rootstocks. Although organic acids were evenly distributed among the evaluated grapes, ‘BRS Carmem’ had the highest sugar levels. The content of phenolic compounds in the grape skin was higher than in the pulp. Overall, ‘BRS Violeta’ surpassed the others, since it presented the highest concentrations of all studied (poly)phenol compounds. AOX decreased in the following order: ‘BRS Violeta’ > ‘IAC 138–22 Máximo’ > ‘BRS Cora’ > ‘BRS Carmem’ > ‘Isabel Precoce’. Despite the rootstocks having little influence on the evaluated compounds, their effect was relevant to ‘BRS Violeta’ grafted on ‘IAC 766’. Finally, the phytochemical profile of grapes ranged according to the variety and rootstock used in cultivation and presents the characterization of Brazilian grape varieties grown on Brazilian rootstocks.

## Supporting information

S1 Graphical abstract (TIF)
