# Supplementary figures and images for: Phytochemical profile of Brazilian grapes (Vitis labrusca and hybrids) grown on different rootstocks
Source: PLoS One. 2022 Oct 20;17(10):e0275489. doi: 10.1371/journal.pone.0275489 (PMC9584379; doi:10.1371/journal.pone.0275489)

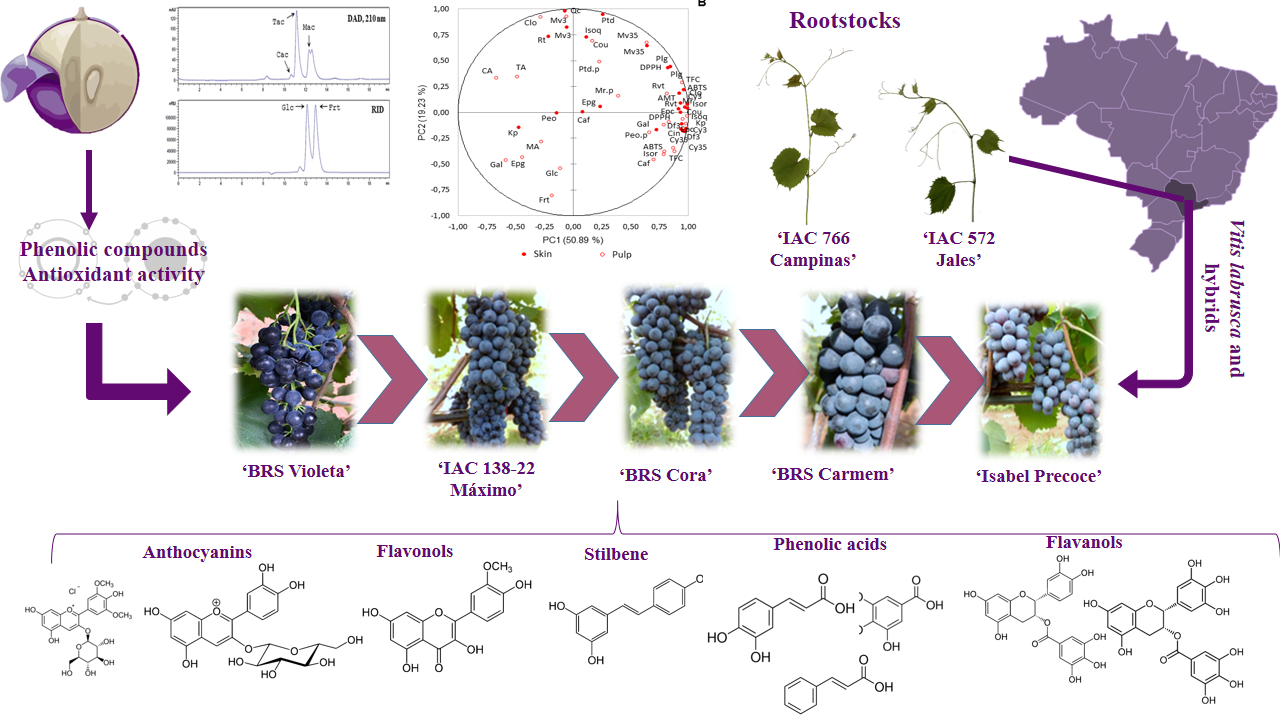

Supplement: S1 Graphical abstract — (TIF) [file pone.0275489.s001.tif]
